# Burnout in hospital staff using partial least squares path modeling for job-person fit: The case of a tertiary referral hospital in southwest Iran

**DOI:** 10.1371/journal.pone.0262774

**Published:** 2022-01-21

**Authors:** Sulmaz Ghahramani, Navid Omidifar, Saghar Garayemi, Mohammad Sayari, Kamran Bagheri Lankarani

**Affiliations:** 1 Health Policy Research Center, Institute of Health, Shiraz University of Medical Sciences, Shiraz, Iran; 2 Research Center of Quran, Hadith and Medicine, Shiraz University of Medical Sciences, Shiraz, Iran; 3 Department of Pathology, Medical School, Shiraz University of Medical Sciences, Shiraz, Iran; Ashkelon Academic College, ISRAEL

## Abstract

Recent studies on burnout (BO) have included both individual and situational factors, referred to as job-person fit (JPF). The present study aimed to evaluate the prevalence rate of BO in the hospital staff working at a tertiary referral hospital in southwest Iran and then to highlight the importance of the person in the context of his/her work life. This cross-sectional study was conducted in 2020 on all hospital staff using a three-part questionnaire comprised of personal and work-situational factors, the Perceived Stress Scale (PSS), and the Psychological Empowerment Scale (PES). The partial least squares (PLS) path modelling and the neural network (NN) model were used to identify the significant variables within the BO dimensions. A total of 358 staff completed the questionnaire and were recruited for the study. Emotional exhaustion (EE) was seen in 137 medical staff (38.3%) and depersonalization (DP) was observed in 75 individuals (20.1%). Thinking about job change was the most important factor positively correlated with EE. Positive stress and work experience were among the most significant factors negatively associated with PA and DP, respectively. The hospital staff experienced BO in a way comparable to the national results. Work-situational and personal variables interacted with the three dimensions of BO in the hospital staff. More experienced staff also felt more accomplished and successful, resulting in the identification of a decreased level of DP and elevated PA.

## 1-Introduction

Burnout (BO) is typically characterised by emotional exhaustion (EE), depersonalization (DP), and lack of personal accomplishment (PA). Although it is not regarded as a disease, affected individuals could potentially seek health care services. BO mainly occurs in people having the most frequent interactions with others, specifically healthcare providers (HCPs). The prevalence rate of BO is relatively high among these individuals. The prevalence rate of BO in nurse groups has been widely studied around the world, including in Iran, wherein reports have shown its effects on more than one-third of nurses [1–7]. At individual levels, BO can shape job satisfaction and job change, and at a broader spectrum, it disturbs nursing productivity, patient health outcomes, patient satisfaction, quality of care, as well as health care system costs [8–12]. Although BO can have an impact on HCPs at various levels, it can influence HCPs differently.

Individual and organisational factors, as well as the quality of job communication and those linked to the end of a patient’s life, are all related to the effect of BO on HCPs [2]. At the individual level, both the personal characteristics of patients and HCPs together with social support are of utmost importance [13]. BO has been identified as the outcome of chronic stress in the workplace that has not been adequately controlled at the organisational level. Developments of BO research have revealed that the given construct has its own complexities, leading researchers to place more focus on the stress faced by individuals within the larger organisational context of people’s relationships to their work [14]. Expanding this construct also results in laying more focus on engagement, namely, the positive antithesis of BO, which can provide new viewpoints on interventions to mitigate BO [14]. The researchers in these studies sought to address two critical questions: first, how BO occurs in the context of an individual work situation and what its correlates are, given that BO is an individual experience that is context-specific (work situation-specific); and second, which personal factors influence different responses to the work situation. Individual characteristics associated with BO include demographic variables, personality characteristics, and work-related attitudes. Though the effect of these elements appears to be less than that of situational factors, others argue that BO is more of a social than an individual phenomenon (personal factors).

Recent work on BO has begun to develop new theoretical frameworks that can more explicitly integrate both individual and situational factors rather than treating them as discrete, the so-called job-person fit (JPF) [15], which aids comprehension of the BO framework and recognises the effect of personal factors on this construct in specific work situations.

In this regard, Maslach and Leiter (1997) first presented six domains, namely, the areas of work-life, whose mismatch would increase BO while a rise in their harmony would lead to job engagement. The Areas of Work-Life Scale (AWS) is thus a model suggesting a structured framework to reflect on the six areas of work-life, including workload, control, reward, community, fairness, and values, which have been reverberated through the literature on BO over the last decades.

Studies in Iran have mostly concentrated on the extent of BO and its effective correlation without considering personal and work-situational factors altogether [4–7] or have not provided a framework for their possible interactions [7]. The AWS had not been cross-culturally validated in the Persian language at the time of the present study, so factors that could best depict the work-situational and personal factors needed to be developed. Several factors were thus extracted from the literature to build up a framework compatible with the hospital context in this study, using structured questions as well as valid and reliable questionnaires to assess these factors (Table 1), and then their hypothesised relationships with the core element of BO and its dimensions were explained.

**Table 1:** The independent variables and their hypothesised relationships with burnout dimensions.

The study framework	Factors as a proxy of job-person fit	Assessment method	Negative Relation to the dimension of burnout*
Personal factors	Demographic variables	Marital status, work experience (year), employment status*, sex and education	All burnout dimensions
	work-related attitudes	offer of own job to their child, second job out of hospital	All burnout dimensions
work situation factors	Workload	Think of a change of job, job shift (fixed or ritational), or engaging with the management of near-death patients	EE, PA
	Control	Empowerment al work scale**, perceived stress***	PA
	Reward and value	Empowerment al work scale, Meaning dimension of Empowerment at work scale	PA
	Community and Fairness	Sufficient time for family and personal life, Absence from work without a good reason	All burnout dimensions

All Burn out dimensions include: EE, DP and, PA.

EE, emotional exhaustion.

DP, depersonalization.

PA: Personal accomplishment.

* Employment status: Stable is defined as employee hired by the government in permanent (stable) vs non- permanent manner (non-stable) including contractual, hired by a company.

** Was assessed by Empowerment al work scale questionnaire and include four dimensions: Impact, meaning, competence, and self-determination.

*** Was assessed by the perceived stress questionnaire.

It is noteworthy to mention that the study was conducted during the Coronavirus disease 2019 (COVID-19) pandemic in which studies show that the BO became more prevalent during the pandemic [16].

The present study aimed to evaluate the prevalence rate of BO in the hospital staff working at a tertiary referral hospital in southwest Iran and to highlight the importance of looking at the person in the context of his/her work life. Then, partial least squares (PLS) path modelling was employed to assess the hypothetical framework of interactions for work-situational and personal factors within the three dimensions of BO in the hospital staff. Ultimately, the neural network (NN) model was utilised for the evaluation of the important factors.

## 2-Materials and methods

### 2-1-Study population

This cross-sectional study was performed on all staff of a tertiary referral hospital in Shiraz, southwest Iran, during May-June of 2020. This hospital was not a referral or backup hospital for the management of COVID-19 patients. There were 587 staff (excluding doctors) with more than 6 months of work experience in this hospital, including medical, administrative, and para-clinic staff working in various wards (herein, units), namely, gynecology, obstetrics, operating and recovery room, neonatal ward, neonatal intensive care unit (ICU), internal medicine, adult ICU, in vitro fertilization (IVF) ward, emergency ward, out-patient clinic, radiology, laboratory, administrative offices, installations, and repairs. The main activities of this centre were providing medical care specific to women’s health, prenatal and postpartum stages, as well as their newborns; most of the hospital staff were women. The levels of BO in this centre had not been studied before. All hospital staff who had worked for at least 6 months in this hospital were included, and staff who have not given consent to participate were excluded.

### 2-2-Study procedure

The study consisted of two phases. In the first phase, the concept of “BO” was valued, which was in itself a step forward. In this way, a person whom the staff trusted most was selected. This person, known as the “trusted employee of the project,” informed the staff about the study in ways specific to the hospital context. These methods included sending letters, phone calls, face-to-face meetings, and so on. Of note, all the units were informed about the study process. This person further documented the information access method. Besides, it was acknowledged that the present study had been designed to identify BO in the staff because their well-being was important and they should not be overly expected within the organization. The staff were also informed about the confidentiality of data and written informed consent was obtained.

In the second phase, all hospital staff were invited to participate in the study. The three dimensions of BO were assessed, and the interaction between work-situational and personal factors with the three dimensions of BO was further identified. Although there were several ways to evaluate employee well-being, due to the lack of an external benchmark in Iran, tools with acceptable validity and reliability were used among all employees in this study. According to the proposed framework appropriate to the hospital environment, the items of the questionnaire for this study were identified. The questionnaire was then distributed among all the staff and collected after a two-day deadline. Several boxes were set up to collect the questionnaires so that the personnel could place them in boxes to ensure the confidentiality and trust of the staff. The deadline was extended for the remaining two days if necessary. The number of questionnaires distributed and collected was documented by the trusted employee of the project. To ensure the representativeness of the study population, hospital units with low response rates (lower than 60%) were again contacted by a trusted employee of the project and encouraged to respond and return the questionnaires. The questionnaires were additionally assessed for the representativeness of age, sex, and unit. This study was also approved by the Ethics committee of Shiraz University of Medical Sciences, Shiraz, Iran, with the ethical code of IR.SUMS.REC.1398.1000.

### 2-3-Instrument of investigation

The questionnaire used in this study generally included three parts. The first part encompassed personal factors, including demographic variables as well as those linked to work attitudes (Table 1). The second part also reflected on work-situational factors along with those related to workload, control, reward, value, community, and fairness (Table 1). For this part, two valid and reliable questionnaires, namely, the Perceived Stress Scale (PSS) and the Empowerment at Work Scale (EWS), were used.

The 14-item PSS was developed by Cohen in 1983, in which each item could be answered based on a Likert-type scale. The PSS could measure two subscales, namely, (a) negative stress and (b) positive stress, which could be scored inversely. Accordingly, a score greater than or equal to 28 was considered to present high levels of stress, and a lower score represented low levels of stress. Of note, the PSS aimed to know how much a person’s life situations were stressful [17], using items with a universal nature and relatively free of content specific to any subpopulation group and acceptable psychometric properties [18].

The EWS also consisted of 12 standard items and four subscales, viz. impact, meaning, competence, and self-determination [19], scored based on a five-point Likert-type scale, from 1 (strongly disagree) to 5 (strongly agree). Higher values could thus indicate higher levels of experienced meaning, competence, self-determination, and impact [20]. Of note, the Persian version of this questionnaire was used in this study [21].

In the final part, BO as the dependent variable was evaluated. As the gold standard for BO assessment, the Persian Version of the Maslach Burnout Inventory-Human Services Survey (MBI-HSS) [22] was used. This 22-item questionnaire had three dimensions, EE, DP, and PA, and was scored from never (zero) to daily (6) (namely, seven points for each item).

The staff could thus express their feelings according to the options available. Higher scores in EE and DP and lower scores in PA could thus demonstrate higher levels of BO. Based on the study by Shanafelt et al. [23], a score greater than or equal to 27 in the EE and/or a score greater than or equal to 10 in the DP could be considered as having at least one manifestation of BO. This scoring method has also been expressed in other studies for HCPs [24, 25].

### 2-4-Statistical analysis

In this study, a two-step procedure was applied to evaluate the effects of the variables related to the BO dimensions (viz. EE, DP, and PA). In this study, PLS path modeling, which is a variance-based structural equation modelling that does not require any distributional assumptions, was used in the first step to identify the significant variables with the BO dimensions [26], and this type of path analysis was also robust to small sample size and complex structural models [27]. In the second step, the variables with significant paths were entered into the NN model to extract their importance [27]. The NN model was thus capable of detecting both linear and non-linear relationships between the independent and dependent variables, a capability not available in PLS path modelling. However, because of the black-box nature of the NN model, it was not capable of testing the relationships [28]. Therefore, the combination of these two procedures created a procedure that was able to test the relationships and had the capability of capturing both linear and non-linear relationships. Moreover, the Kolmogorov-Smirnov test was applied to assess the normality of the dependent variables. The hospital units were further categorised into non-medical and medical ones, based on whether they were involved in direct diagnostic or therapeutic service delivery to patients or not. Mann-Whitney U test was additionally employed to assess the hypothesis of no difference across the hospital units (non-medical vs. medical) in terms of BO dimensions. The significance level was set at 0.05.

## 3-Results

### 3-1-Description of sample characteristics

More than 60% of the staff returned the completed questionnaires (= 358). The independent variables and their hypothesisted relationships with BO dimensions are summarised in Table 1. Tables 2 and 3 present the subgroups of the qualitative variables and their frequencies, as well as the common descriptive statistics for the quantitative variables. The mean age of the staff was also 34.6± 7.7 years old. As listed in Table 2, 23 participants (6.4%) were male and 80 cases (22.3%) were unmarried. In addition, 241 participants (67.3%) had rotational work shifts. EE was also seen in 137 cases (38.3%) and DP was observed in 75 individuals (20.1%). As well, EE and DP were concurrently reported in 54 participants (15.1%). The distribution of the BO dimensions in different hospital units (medical vs. non-medical) is shown in Table 2.

**Table 2:** Frequency of qualitative variables (n = 358).

qualitative variables			
Variable	Subgroups	Frequency	Percent
Gender	Male	23	6.4
	Female	332	92.7
	Missing	3	.8
Marital status	Unmarried	80	22.3
	Married	256	71.6
	Missing	22	6.1
Education	Diploma or less education	22	6.2
	Associate and bachelor’s degree	258	72.1
	Master’s degree or higher	32	9
	Missing	46	12.8
Job shift	Fixed	100	27.9
	Rotational	241	67.3
	Missing	17	4.7
Employment status*	Non-stable	110	30.7
	Stable	226	63.2
	Missing	22	6.1
Enough time for family/personal life (free time)	Don’t have	65	18.2
	Have	279	77.9
	Missing	14	3.9
Absence from work without a good reason(absent)	Don’t have	324	90.5
	Have	15	4.2
	Missing	19	5.3
Think of change of job (job change)	No	159	44.4
	Yes	182	50.8
	Missing	17	4.7
offer of own job to the child** (job offer)	No	165	46.1
	Yes	135	37.7
	Missing	58	16.2
Second job out of hospital	Don’t have	329	91.9
	Have	13	3.6
	Missing	16	4.5
Engaged with the management of near dying patients	No	236	65.9
	Yes	62	17.3
	Missing	60	16.8
Work unit	Medical	232	64.8
	Non-medical	122	34.1
	Missing	4	1.1
Job	Nurse	139	38.8
	Nurse assistant	10	2.8
	Midwife	83	23.2
	Other staff	122	34.1
	Missing	4	1.1
Emotional exhaustion***	Medical units	95	40.1
	Non-medical units	42	34.4
	Overall	137	38.3
Depersonalization***	Medical units	54	23.3
	Non-medical units	21	17.2
	Overall	75	20.1

* Employment Status: Stable is defined as an employee hired by the government in a permanent (stable) vs non-permanent manner (non-stable) including contractual, hired by a company…

** Would you recommend your children pursue your career?

*** a score greater than or equal to 27 in emotional exhaustion and/or a score of 10 or higher in depersonalization scale indicates as having at least one manifestation of BO.

**Table 3:** Common descriptive statistics for quantitative variables (n = 358).

quantitative variables					
Variable	Number of valid observations	Minimum	Maximum	Mean	Std. Deviation
Emotional exhaustion*	354	0	54	22.66	12.48
Personal accomplishment*	354	0	47	27.05	8.12
Depersonalization*	354	0	24	5.63	5.51
Work experience	288	0	40	11.65	7.50
Competence**	349	3	15	12.13	2.54
Self-determination**	349	3	15	8.57	2.97
Impact **	349	1	15	9.49	2.75
Meaning **	349	3	15	11.59	2.76
Negative stress***	352	0	28	13.19	5.43
Positive stress***	352	4	25	13.67	3.43

*Burn-out dimensions include emotional exhaustion, depersonalization, and personal accomplishment.

** ** The dimensions of the Empowerment at Work Scale questionnaire include impact, meaning, competence, and self-determination.

*** The dimensions of the perceived stress questionnaire include negative stress and positive stress.

Concerning the missing variables shown in Tables 2 and 3, data from 189 employees was used in the PLS path modelling and the NN model. The characteristics of these 189 staff are described in Table A in S1 File. The results of the Kolmogorov-Smirnov test also indicated the non-normal distribution of the BO dimensions (Table B in S1 File).

### 3-2-PLS path modeling

Since the dependent variables in this study were not normally distributed, the PLS path modelling was performed using the SmartPLS 3.0 software. The goodness of fit (GoF) was further evaluated through the standardised root mean square residual (SRMR), wherein the SRMR value of less than 0.8 revealed an acceptable fit [29]. The t-statistics and the p-values of the path coefficients were correspondingly estimated via bootstrapping [30]. The results of the PLS path modelling for the significant paths are illustrated in Table 4. The results of the Mann-Whitney U test also demonstrated no statistically significant difference between the medical and non-medical units in terms of the BO dimensions, so the results were not separated based on the units (Table C in S1 File). Moreover, the SRMR value was 0.04, suggesting an acceptable fit to the model. For the EE dimension, the results implied that offering one’s job to their children, having stable employment status, and rotational work shifts were negatively and significantly related, and thinking of job change was positively and significantly associated with EE. Meaning and work experience were also positively and significantly related to the PA dimension, but positive stress was negatively associated with PA. Considering the DP dimension, the female participants and those with more work experience and more free time for family and personal life had less DP. The complete results of the PLS path modelling are provided in Tables D–F in S1 File.

**Table 4:** PLS path modeling results for significant paths.

Paths form	Paths to	Path coefficients	Standard deviation	T-statistics	P-values
Job change (yes)	Emotional Exhaustion	0.494	0.051	9.769	<0.001
Job offer (yes)		-0.19	0.065	2.911	0.004
Employment status (stable)		-0.252	0.088	2.86	0.015
Job shift (rotational)		-0.146	0.07	2.086	0.029
Meaning	Personal accomplishment	0.279	0.093	2.993	0.003
Work experience		0.191	0.093	2.056	0.041
Positive stress		-0.25	0.086	2.906	0.033
Work experience	Depersonalization	-0.292	0.098	2.966	0.003
Gender (female)		-0.232	0.106	2.186	0.03
Free time (have)		-0.207	0.08	2.5875	0.039

### 3-3-NN model

In this research, the NN procedure with multilayer perceptron was performed using the SPSS Statistics software (ver. 26). The predictors mentioned in Table 4 were also entered into the model as inputs, and the BO dimensions (EE, DP, and PA) were considered as outputs in the respective models. Ten-fold cross-validation, which could split the dataset into 90% for the training data and 10% for the testing data, was further carried out to assess the accuracy of the models. The Root Mean Square Error (RMSE) values were also estimated for training and testing the datasets for 10 networks in each model [27]. The RMSE values are provided in Table G in S1 File. The consistent values of RMSE for training and testing indicated reliable fits for the three models. In Table 5, the importance of the input variables in terms of normalised importance is presented. For EE, job change was the most significant variable, followed by a job offer. Considering PA, positive stress was the key variable, followed by work experience. For DP, work experience was the most significant predictor, followed by gender.

**Table 5:** NN analysis to assign the importance of variables.

Dependent variable	Independent Variable	Importance	Normalized Importance
Emotional exhaustion	Job change (yes)	0.651	100.00%
	Job offer (yes)	0.218	33.50%
	Job shift (rotational)	0.086	13.20%
	Employment status (stable)	0.045	6.90%
Personal accomplishment	Positive stress	0.475	100.00%
	Work experience	0.297	62.70%
	Meaning	0.228	48.00%
Depersonalization	Work experience	0.59	100.00%
	Gender (female)	0.267	45.30%
	Free time (have)	0.143	24.30%

## 4-Discussion

Based on the definition of high scores on the EE or DP subscales as having at least one manifestation of BO [23, 31], EE was seen in 137 cases (38.3%) and DP was observed in 75 participants (20.1%). EE and DP were concurrently reported in 54 individuals (15.1%). This study revealed that non-medical hospital staff might experience BO similar to medical ones, although at lower levels. This has also been evidenced that all hospital staff might experience BO [32].

In this study, 40.1% of the medical staff (namely, nurses, nurse assistants, and midwives), comprising more than 64% of the participants, experienced EE. The overall prevalence of BO was estimated to be 36% in a meta-analysis of 4180 Iranian nurses [6]. The prevalence rate of BO in nurses working in ICUs, emergency wards, and oncology, as well as palliative wards, also demonstrated similar results [1–3, 33]. The emergence of the COVID-19 and its consequences in hospital settings has resulted in changes in the prevalence of BO among HCPs [34]. The hospital included in this study was not a frontline hospital for COVID-19 cases. Nonetheless, it is evident that even the non-frontline COVID-19 exposed HCPs might also experience high BO prevalence during the COVID-19 pandemic [16].

Through the PLS path modelling in this study, with an acceptable fit to the model, the findings showed that work-situational as well as personal variables interacted with the three dimensions of BO in the hospital staff, in line with previous research wherein four groups of characteristics, including individual and organisational ones, along with the quality of job communication and factors related to the end of a patient’s life, had BO based on the multivariate analysis (MVA) [2].

This study also showed that thinking about job change was positively related to EE while the staff who offered their jobs to their children, had stable employment status, and rotational work shifts experienced lower EE. These factors are categorised as personal and workload in the framework concerned (Table 1). The direct relationship between workload and EE has been evidenced earlier [35]. Among these factors, thinking of job change was the most important factor related to EE. However, it had been supposed that DP was the key dimension, finally leading to job turnover [35], but it seems that EE was associated with thinking about a job change at the earlier stages. Moreover, this was in agreement with a national study in France on ICU nurses, reporting thoughts of changing jobs, which was significantly higher among nurses who experienced severe BO compared with their counterparts [2].

The second more important factor related to EE was the job offer, which was phrased as “Do you recommend your children pursue your career?” to give a picture of the attitude of the staff toward their work. Generally, only half of the surgeons in the United States (with a prevalence rate of 40% BO) had recommended their careers to their children [36]. Moreover, the study findings revealed that the staff with rotational work shifts had lower EE. This might be due to a lower workload during night shifts in this hospital setting or more diversity in work schedules or the staff communicating with them.

Positive stress was also negatively correlated with PA in the hospital staff. Accordingly, the staff with higher work experience and higher scores of meaning (namely, EWS dimensions) had better PA. These factors were once again compatible with the personal factor, control, reward, and value in the proposed framework. Positive stress and work experience were the most important factors related to PA. The relationship between stress and DP has also been established [37]. The positive relationship between stress and BO was additionally stated for PA as well as EE dimensions [38]. This difference could be attributed to the discrepancy in the context of the study, which could in turn influence stress.

It is important to highlight the negative effects of chronic stress in the workplace, which have not been managed correctly. This showed that stress and BO had also led to poorer perceived control [39]. Management strategies for stress reduction might be holistic and should not be limited to individual strategies. In this sense, organisations need to provide resources that make it easier for the staff to make the best use of available individual strategies to prevent BO. Unfortunately, most healthcare centres merely focus on individual interventions. Whenever suggestions are person-centered and not supplemented by authentic efforts to address system-based issues that lead to BO, such as workload, the staff might be indifferent or pessimistic in response to interventions.

More work experience, female gender, and more free time for family and personal life were all found to be negatively associated with DP. These were harmonious with personal factors, community, and fairness in the given framework.

Work experience, as a personal factor, was also positively and negatively associated with PA and DP, respectively. As well, work experience had been supposed to be a risk factor for the BO of ICU nurses in a systematic review, though these findings had not been consistent in the studies included [40]. Higher work experience could be both protective and predictive for BO, and this seems to be related to the specific work environment. The findings of the present study rationalise that more experienced staff can grow through their experiences and develop into skilled individuals dedicated to their work; therefore, feeling more accomplished and successful would result in the identification of a decreased level of DP and increased PA.

Understanding modifiable and non-modifiable personal and work-situational factors affecting BO can be useful for hospital policy-makers to plan for BO-reducing interventions. These interventions could be performed at different individual and organisational levels as well as in work situations [13, 41]. Further studies to understand appropriate context-specific interventions, prioritisation of the interventions, and the effectiveness of the interventions to decrease BO are suggested.

This study highlighted personal as well as work-situational factors that can have an impact on each dimension of BO and confirmed the importance of each factor using the NN model. It is unclear whether the staff with BO are less likely to complete surveys due to apathy or more likely to complete them because of more attention to this issue. In addition, this study was cross-sectional and was not capable of defining causal relationships. No doubt, there were features related to BO that were not measured in this study. Furthermore, the results could vary by subspecialty, geographic region, and the practise environment.

## 5-Conclusions

Both medical and non-medical staff in this study experienced BO in a way comparable to the national results. Work-situational and personal factors also interacted with the three dimensions of BO in the hospital staff. Besides, thinking about job change and stress were the most important factors related to EE and PA, respectively. More experienced staff also felt more accomplished and successful, resulting in the identification of a decreased level of DP and elevated PA.

## Supporting information

S1 File Supplementary results. (DOCX)

S1 Dataset The minimal anonymized data. (XLSX)

## Abbreviations

BO: Burnout
EE: emotional exhaustion
PA: Personal accomplishment
DP: depersonalization
PLS: partial least squares
NN: neural network
JPF: job-person fit
HCPs: health care providers
IVF: in vitro fertilization
PSS: Perceived Stress Scale
EWS: Empowerment at Work Scale
MBI-HSS: Maslach Burnout Inventory-Human Services Survey
GoF: the goodness of fit
SRMR: standardized root mean square residual
RMSE: Root Mean Square Error
MVA: multivariate analysis
ICU: intensive care unit
