# Supplementary material for: Burnout in hospital staff using partial least squares path modeling for job-person fit: The case of a tertiary referral hospital in southwest Iran
Source: PLoS One. 2022 Jan 21;17(1):e0262774. doi: 10.1371/journal.pone.0262774 (PMC8782409; doi:10.1371/journal.pone.0262774)
Supplement: S1 File — (DOCX) [file pone.0262774.s001.docx]

**S1 File:** **Supplementary results**

**Supplement to: Burnout in Hospital Staff Using Partial Least Squares Path Modeling for Job -Person Fit: The Case of a Tertiary Referral Hospital in Southwest Iran**

**Table A. The frequency of qualitative variables and common descriptive statistics for quantitative variables for Partial least squares path analysis and Neural network analysis (N=189).**

| **qualitative variables** | | | | | | |
| --- | --- | --- | --- | --- | --- | --- |
| **Variable** | **Subgroups** | | | **Frequency** | | **Percent** |
| Gender | Male | | | 11 | | 5.8 |
|  | Female | | | 178 | | 94.2 |
| Marital status | Unmarried | | | 43 | | 22.8 |
|  | Married | | | 146 | | 77.2 |
| Education | diploma or less education | | | 12 | | 6.3 |
|  | Associate and bachelor's degree | | | 152 | | 80.4 |
|  | master's degree or higher | | | 25 | | 13.2 |
| Job shift | Fixed | | | 60 | | 31.7 |
|  | Turning | | | 129 | | 68.3 |
| Employment status | non-stable | | | 116 | | 61.4 |
|  | Stable | | | 73 | | 38.6 |
| enough time for family/personal life (free time) | don't have | | | 31 | | 16.4 |
|  | Have | | | 158 | | 83.6 |
| Absence from job without any important cause (absent) | don't have | | | 182 | | 96.3 |
|  | Have | | | 7 | | 3.7 |
| Think of change of job (job change) | No | | | 82 | | 43.4 |
|  | Yes | | | 107 | | 56.6 |
| offer of own job to the child (job offer) | No | | | 106 | | 56.1 |
|  | Yes | | | 83 | | 43.9 |
| second job out of hospital | don't have | | | 182 | | 96.3 |
|  | Have | | | 7 | | 3.7 |
| engaged with the management of near dying patients | No | | | 146 | | 77.2 |
|  | yes | | | 43 | | 22.8 |
| **quantitative variables** | | | | | | |
| Variable | Minimum | Maximum | Mean | | Std. Deviation | |
| Emotional exhaustion^*^ | 1 | 52 | 22.635 | | 12.501 | |
| Personal accomplishment ^*^ | 7 | 47 | 27.984 | | 7.794 | |
| Depersonalization ^*^ | 0 | 24 | 5.503 | | 5.586 | |
| Work experience | 0 | 31 | 10.905 | | 7.058 | |
| Competence ^**^ | 5 | 15 | 12.519 | | 2.285 | |
| Self-determination^**^ | 3 | 15 | 8.624 | | 2.897 | |
| Impact ^**^ | 1 | 15 | 9.556 | | 2.922 | |
| Meaning ^**^ | 4 | 15 | 12.021 | | 2.570 | |
| Negative stress^***^ | 3 | 28 | 13.577 | | 5.443 | |
| Positive stress^***^ | 6 | 23 | 13.492 | | 3.311 | |
| *Burn out dimensions include: emotional exhaustion, depersonalization and Personal accomplishment.  ** Empowerment al work scale questionnaire dimensions include:impact, meaning, competence, and self-determination.  *** Perceived stress questionnaire dimensions include: Negative stress and Positive stress. | | | | | | |

**Table B. The results of Kolmogorov-Smirnov test.**

|  | Inefficacy | Exhaustion | Cynicism |
| --- | --- | --- | --- |
| Test Statistic | .069 | .091 | .183 |
| P-value ^a^ | .029 | .001 | <0.001 |
| a. Values less than 0.05 reject the normality | | | |

**Table C. The results of Mann-Whitney U test.**

|  | Statistic value | Z | P-value ^a^ |
| --- | --- | --- | --- |
| Emotional exhaustion | 3650.5 | -0.982 | 0.326 |
| Personal accomplishment | 3781 | -0.616 | 0.538 |
| Depersonalization | 3886 | -0.322 | 0.748 |
| 1. Values more than 0.05 indicate no difference between medical and non-medical units | | | |

**Table D. The results of PLS path analysis (emotional exhaustion).**

| Paths form | Paths to | Path coefficients | Standard deviation | T-statistics | P-values |
| --- | --- | --- | --- | --- | --- |
| Job change(yes) | Emotional Exhaustion | 0.494 | 0.051 | 9.769 | <0.001 |
| Job offer(yes) |  | -0.19 | 0.065 | 2.911 | 0.004 |
| Employment status (stable) |  | -0.252 | 0.088 | 2.86 | 0.015 |
| Job shift (turning) |  | -0.146 | 0.07 | 2.086 | 0.029 |
| Gender(female) |  | -0.168 | 0.095 | 1.76 | 0.08 |
| ﻿ Marital status (married) |  | 0.069 | 0.064 | 1.08 | 0.281 |
| near dying patients (yes) |  | -0.04 | 0.064 | 0.619 | 0.536 |
| Education (master's degree or higher) |  | -0.023 | 0.1 | 0.233 | 0.816 |
| Education (Associate and bachelor's degree) |  | 0.01 | 0.1 | 0.099 | 0.921 |
| Work experience |  | -0.003 | 0.091 | 0.037 | 0.971 |
| second job(have) |  | 0.001 | 0.081 | 0.012 | 0.997 |

**Table E. The results of PLS path analysis (Personal accomplishment).**

| Paths form | Paths to | Path coefficients | Standard deviation | T-statistics | P-values |
| --- | --- | --- | --- | --- | --- |
| Meaning | Personal accomplishments | 0.279 | 0.093 | 2.993 | 0.003 |
| Work experience |  | 0.191 | 0.093 | 2.056 | 0.041 |
| Positive stress |  | -0.25 | 0.086 | 2.906 | 0.033 |
| Education (Associate and bachelor's degree) |  | -0.188 | 0.138 | 1.366 | 0.174 |
| Negative stress |  | -0.109 | 0.083 | 1.314 | 0.191 |
| Education (master's degree or higher) |  | -0.181 | 0.14 | 1.291 | 0.198 |
| second job(have) |  | 0.101 | 0.082 | 1.237 | 0.218 |
| impact |  | 0.09 | 0.086 | 1.051 | 0.294 |
| Job offer (yes) |  | -0.031 | 0.068 | 0.459 | 0.647 |
| Gender (female) |  | 0.031 | 0.071 | 0.442 | 0.659 |
| Employment status (stable) |  | 0.038 | 0.097 | 0.388 | 0.699 |
| competence |  | 0.019 | 0.084 | 0.222 | 0.825 |
| Self determination |  | 0.015 | 0.081 | 0.184 | 0.854 |
| ﻿ Marital status (married) |  | -0.002 | 0.08 | 0.02 | 0.984 |

**Table F. The results of PLS path analysis (Depersonalization).**

| Paths form | Paths to | Path coefficients | Standard deviation | T-statistics | P-values |
| --- | --- | --- | --- | --- | --- |
| Work experience | Depersonalization | -0.292 | 0.098 | 2.966 | 0.003 |
| Gender (female) |  | -0.232 | 0.106 | 2.186 | 0.03 |
| Free time (have) |  | -0.207 | 0.08 | 2.5875 | 0.039 |
| Job offer (yes) |  | -0.084 | 0.069 | 1.225 | 0.222 |
| Education (Associate and bachelor's degree) |  | -0.145 | 0.119 | 1.216 | 0.225 |
| Employment status (stable) |  | 0.063 | 0.098 | 0.64 | 0.523 |
| Second job (yes) |  | -0.052 | 0.093 | 0.559 | 0.576 |
| Absence from job (have) |  | -0.02 | 0.05 | 0.387 | 0.699 |
| ﻿ Marital status (married) |  | 0.023 | 0.08 | 0.287 | 0.774 |
| Education (master's degree or higher) |  | -0.04 | 0.142 | 0.283 | 0.778 |

**Table G. RMSE for neural network.**

|  | Output: Exhaustion | | Output: Inefficacy | | Output: Cynicism | |
| --- | --- | --- | --- | --- | --- | --- |
| Network | Training | Testing | Training | Testing | Training | Testing |
|  | 0.809 | 0.859 | 1.252 | 1.382 | 0.977 | 1.184 |
|  | 0.823 | 0.744 | 1.246 | 1.306 | 1.003 | 0.995 |
|  | 0.826 | 0.671 | 1.296 | 0.950 | 0.978 | 0.919 |
|  | 0.820 | 0.845 | 1.224 | 1.315 | 0.953 | 1.008 |
|  | 0.820 | 0.736 | 1.242 | 1.340 | 0.962 | 1.103 |
|  | 0.806 | 0.974 | 1.023 | 1.007 | 0.952 | 1.075 |
|  | 0.785 | 1.042 | 1.246 | 1.255 | 0.998 | 0.959 |
|  | 0.794 | 0.978 | 1.204 | 1.143 | 0.963 | 0.901 |
|  | 0.807 | 0.917 | 1.216 | 1.460 | 0.936 | 1.125 |
|  | 0.808 | 0.867 | 1.259 | 0.945 | 0.983 | 0.867 |
| Mean | 0.809 | 0.863 | 1.221 | 1.210 | 0.971 | 1.013 |
| Std. Deviation | 0.0132 | 0.119 | 0.074 | 0.187 | 0.021 | 0.105 |
